# The effect of breed and diet type on the global transcriptome of hepatic tissue in beef cattle divergent for feed efficiency

**DOI:** 10.1186/s12864-019-5906-8

**Published:** 2019-06-26

**Authors:** Marc G. Higgins, David A. Kenny, Claire Fitzsimons, Gordon Blackshields, Séan Coyle, Clare McKenna, Mark McGee, Derek W. Morris, Sinéad M. Waters

**Affiliations:** 1 Discipline of Biochemistry, National University of Ireland, Galway, Ireland; 2 Animal and Bioscience Research Department, Animal & Grassland Research and Innovation Centre Teagasc, Grange, Dunsany, Co. Meath, Ireland; 3 Livestock Systems Research Department, Animal & Grassland Research and Innovation Centre Teagasc, Grange, Dunsany, Co. Meath, Ireland; 4 Present address: Department of Agriculture, Fisheries and the Marine, Celbridge, Co. Kildare Ireland

**Keywords:** Bovine genetics, RNA-Seq, Feed efficiency

## Abstract

**Background:**

Feed efficiency is an important economic and environmental trait in beef production, which can be measured in terms of residual feed intake (RFI). Cattle selected for low-RFI (feed efficient) have similar production levels but decreased feed intake, while also emitting less methane. RFI is difficult and expensive to measure and is not widely adopted in beef production systems. However, development of DNA-based biomarkers for RFI may facilitate its adoption in genomic-assisted breeding programmes. Cattle have been shown to re-rank in terms of RFI across diets and age, while also RFI varies by breed. Therefore, we used RNA-Seq technology to investigate the hepatic transcriptome of RFI-divergent Charolais (CH) and Holstein-Friesian (HF) steers across three dietary phases to identify genes and biological pathways associated with RFI regardless of diet or breed.

**Results:**

Residual feed intake was measured during a high-concentrate phase, a zero-grazed grass phase and a final high-concentrate phase. In total, 322 and 33 differentially expressed genes (DEGs) were identified across all diets for CH and HF steers, respectively. Three genes, *GADD45G*, *HP* and *MID1IP1,* were differentially expressed in CH when both the high-concentrate zero-grazed grass diet were offered. Two canonical pathways were enriched across all diets for CH steers. These canonical pathways were related to immune function.

**Conclusions:**

The absence of common differentially expressed genes across all dietary phases and breeds in this study supports previous reports of the re-ranking of animals in terms of RFI when offered differing diets over their lifetime. However, we have identified biological processes such as the immune response and lipid metabolism as potentially associated with RFI divergence emphasising the previously reported roles of these biological processes with respect to RFI.

**Electronic supplementary material:**

The online version of this article (10.1186/s12864-019-5906-8) contains supplementary material, which is available to authorized users.

## Background

Feed provision accounts for more than 70% of direct costs in beef production systems [1]. Selection of feed efficient cattle would improve profits by reducing expenditure on feed while maintaining output [2]. Moreover, there is increasing pressure on the global agri-food industry to improve its environmental footprint, while increasing output to meet the growing demand for protein [3]. Selection for feed efficient cattle could maintain output while concurrently decreasing methane emissions, as it has been suggested that low-RFI beef cattle emit less methane than their inefficient counterparts [4].

Feed efficiency has several methods of measurement including residual feed intake (RFI) [5], which is defined as the difference between an animal’s actual and predicted feed intake. Residual feed intake has gained popularity as a measure of feed efficiency due to its moderate heritability and its phenotypic independence from production traits [2]. It has been suggested that variation in RFI may be due to differences in an animal’s physiological processes, such as those that occur in the liver [6]. The liver is a major metabolic organ in ruminants, typically consuming 24% of total energy [7]. The liver distributes nutrients to organs for both maintenance and production, amongst other functions such as gluconeogenesis [8]. The liver also plays a role in physiological processes such as the immune response, glucose metabolism and lipid metabolism [9, 10]. Due to the multifactorial role of the ruminant liver, any variation in its gene expression may reflect divergent efficiency of overall metabolic and physiological function potentially leading to phenotypic differences in RFI.

Incorporating RFI into breeding programmes would enable selection of feed efficient cattle, thereby improving farm profits. The calculation of RFI requires an expensive and often labour intensive performance measurement period during which individual feed intake and weight gain are recorded for each animal [11]. Residual feed intake’s observed heritability, with an estimated range of 0.26–0.54 [2], has led to considerable international interest in the discovery of accurate and robust biological markers of RFI or other means of identifying low-RFI cattle, such as by using genomic estimated breeding values (GEBVs) or single-step genomic prediction) [12, 13]. However, the use of GEBVs or single-step genomic prediction shed little light on the underlying biology of RFI.

Differences in breed [14, 15] and physiological ages [16, 17], as well as genotype-by-environment interactions, have been observed to cause re-ranking of cattle for RFI status [18, 19]. This represents a challenge in elucidating the underlying biology of RFI as re-ranking of cattle for RFI across diets may indicate that diet causes variation in the biological processes underlying RFI [20]. Therefore, it is important to investigate the biological mechanisms underpinning RFI-divergence across physiological age, breed and diet in order to gain a complete understanding of the biology underpinning this trait.

RNA-Seq, a method by which all expressed genes within a tissue are profiled [21], has been used to identify differentially expressed genes (DEGs) associated with RFI. RNA-Seq offers several advantages over other transcriptome profiling methods including that the technology facilitates the entire transcriptome of an organism to be investigated rather than known genes as is the case for microarray analysis or real time PCR [21]. RNA-Seq analyses have been conducted to investigate variation in gene expression between RFI-divergent cattle in several tissues including liver [22, 23], skeletal muscle [24] and rumen epithelial tissue [25]. Recently, Mukiibi et al.*,* (2018) observed five DEGs across three breeds of Canadian cattle offered the same diet [26].

To identify genes associated with RFI across breed, diet and physiological age, we conducted RNA-Seq analysis of the liver transcriptome of two breeds of cattle subjected to three dietary regimens: a high-concentrate diet, a zero-grazed grass diet and cattle were finished on a high-concentrate diet. The aims of this study were: (i) to elucidate the underlying biology of RFI by investigating key genes and pathways implicated in RFI divergence and (ii) to identify genes and biological functions associated with RFI across multiple breeds and dietary phases in order to highlight candidate genes for further interrogation as potential biomarkers for RFI.

## Results

### Animal model

Across all three dietary phases and within breed, cattle were ranked in terms of RFI and divided into thirds. The steers with the lowest-RFI values were deemed to be low RFI, while those with the highest RFI values were designated to be high RFI. High RFI steers consumed more feed on average than their low RFI counterparts (*P* < 0.001), while having a similar average daily gain (ADG) (*P* > 0.05). As expected, within breed and dietary phase no statistically significant difference in metabolic body weight (MBW) and ADG was observed between the two RFI groups (Table 1). Similar patterns are observed when the animals for which RNA-Seq libraries were generated (Table 2), however the high RFI CH steers offered the zero-grazed grass (ZG) diet displayed a trend to consume less feed than their low RFI counterparts (*P* = 0.07).

**Table 1:** Feed intake, RFI and growth traits for the entire population of low and high RFI steers during different dietary phases

Trait	Diet-Breed	Low (S.D.)	High (S.D.)	*P-*value
DMI (kg/d)	H1.CH	7.8 (0.68)	9 (0.58)	< 0.001
	H1.HF	8.3 (0.87)	9.3 (0.68)	< 0.001
	ZG.CH	8.8 (0.48)	9.4 (0.42)	< 0.001
	ZG.HF	9.1 (0.47)	10 (0.48)	< 0.001
	H2.CH	10.8 (0.69)	12.3 (0.90)	< 0.001
	H2.HF	11.6 (1.18)	13.6 (1.25)	< 0.001
RFI (kg DM/d)	H1.CH	−0.5 (0.15)	0.56 (0.17)	< 0.001
	H1.HF	−0.5 (0.27)	0.53 (0.26)	< 0.001
	ZG.CH	−0.35 (0.16)	0.35 (0.17)	< 0.001
	ZG.HF	−0.42 (0.27)	0.56 (0.18)	< 0.001
	H2.CH	−0.75 (0.29)	0.76 (0.39)	< 0.001
	H2.HF	−1.01 (0.54)	1.03 (0.28)	< 0.001
ADG (kg)	H1.CH	1.3 (0.36)	1.4 (0.26)	0.43
	H1.HF	1.4 (0.35)	1.4 (0.20)	0.78
	ZG.CH	1.4 (0.14)	1.4 (0.22)	0.96
	ZG.HF	1.2 (0.18)	1.3 (0.24)	0.85
	H2.CH	1.4 (0.25)	1.4 (0.28)	0.64
	H2.HF	1.3 (0.47)	1.3 (0.16)	0.98
MBW (kg)	H1.CH	95 (5.15)	96 (5.77)	0.79
	H1.HF	81 (7.81)	80 (6.12)	0.90
	ZG.CH	116 (7.26)	115 (6.41)	0.80
	ZG.HF	102 (3.34)	104 (8.34)	0.90
	H2.CH	139 (6.91)	140 (7.28)	0.79
	H2.HF	131 (8.41)	141 (9.50)	0.90

*DMI* dry matter intake, *RFI* residual feed intake, *ADG* average daily gain, *MBW* metabolic body weight, *CH* Charolais, *HF* Holstein-Friesian, *Low* low RFI, *High* high RFI, *S.D*. Standard deviation, *H1* high concentrate diet 1, *H2* high concentrate diet 2, *ZG* zero-grazed grass diet

**Table 2:** Feed intake, RFI and growth traits for the low and high RFI steers for which RNA-Seq libraries were successfully generated during different dietary phases

Trait	Diet-Breed	Low	High	*P-*value
DMI (kg/d)	H1.CH	7.5 (*n* = 9)	9.1 (*n* = 11)	< 0.001
	H1.HF	7.7 (*n* = 7)	9.7 (n = 9)	< 0.001
	ZG.CH	8.4 (*n* = 3)	9.7 (n = 3)	0.07
	ZG.HF	8.8 (*n* = 10)	10.3 (*n* = 8)	< 0.001
	H2.CH	10.5 (n = 9)	12.9 (n = 8)	< 0.001
	H2.HF	10.8 (*n* = 5)	13.6 (n = 8)	0.002
RFI (kg DM/d)	H1.CH	−0.8	0.8	< 0.001
	H1.HF	−0.9	0.8	< 0.001
	ZG.CH	−0.5	0.5	< 0.001
	ZG.HF	−0.7	0.7	< 0.001
	H2.CH	−1.1	1.2	< 0.001
	H2.HF	−1.9	1.3	< 0.001
ADG (kg)	H1.CH	1.3	1.3	0.9
	H1.HF	1.4	1.2	0.2
	ZG.CH	1.3	1.3	0.6
	ZG.HF	1.3	1.2	0.2
	H2.CH	1.4	1.4	0.8
	H2.HF	1.3	1.4	0.7
MBW (kg)	H1.CH	95	95	0.76
	H1.HF	81	80	0.59
	ZG.CH	113	118	0.53
	ZG.HF	105	104	0.55
	H2.CH	137	142	0.45
	H2.HF	130	127	0.56

*DMI* dry matter intake, *RFI* residual feed intake, *ADG* average daily gain, *MBW* metabolic body weight, *CH* Charolais, *HF* Holstein-Friesian, *Low* low RFI, *High* high RFI, *H1* high concentrate diet 1, *H2* high concentrate diet 2, *ZG* zero-grazed grass diet

### Differential gene expression analysis

A total of 160, 158 and 4 genes (adjusted *P* < 0.1) were identified as differentially expressed between high and low RFI Charolais (CH) cattle for the high-concentrate phase 1 (H1), ZG and high-concentrate phase 2 (H2) diets, respectively. For the Holstein-Friesian (HF) steers; 26, 2 and 5 (adjusted *P* < 0.1) were differentially expressed between RFI cohorts for H1, ZG and H2, respectively. The top DEGs for each comparison are represented in Tables 3 and 4 for CH and HF, respectively. All DEGs for each breed and diet are listed in Additional file 2.

**Table 3:** The most significantly differentially expressed genes between high and low RFI Charolais steers across three dietary phases

Diet and Breed	Gene	LogFC	*P*-value
CH.H1	*TNFAIP3*	0.66	0.0002
	*KRBA1*	1.39	0.00069
	*SIK1*	1.33	0.0043
	*IRS2*	1.23	0.0043
CH.ZG	*SLC39A4*	−2.68	4.58E-09
	*BHMT2*	1.26	7.60E-08
	*TNC*	2.02	2.95E-07
	*ENSBTAG00000016032*	1.58	2.95E-07
	*ABCA6*	1.16	0.0001
CH.H2	*LOC768255*	−3.55	0.00024
	*GIMAP4*	−3.08	0.0065

*RFI* residual feed intake, *CH* Charolais, *H1* high concentrate diet 1, *H2* high concentrate diet 2, *ZG* zero-grazed grass diet, *LogFC* log_2_fold-change in low-RFI steers compared to high-RFI steers; *P*-value = Benjamini-Hochberg corrected *P*-value to account for multiple testing

**Table 4:** The most significantly differentially expressed genes between high and low RFI Holstein-Friesian steers across three dietary phases

Diet and breed	Gene	LogFC	*P-*value
HF.H1	*SNRPD3*	−0.37	0.0010
	*AK3*	−0.35	0.0013
	*GSTM1*	−0.87	0.0061
	*MOB3B*	0.72	0.0061
	*LOC782233*	−5.45	0.0061
	*HPRT1*	−0.33	0.011
	*ENSBTAG00000032859*	−0.66	0.016
	*ACMSD*	−1.04	0.024
	*PARM1*	1.15	0.024
	*ANPEP*	1.10	0.024
	*GUCY2D*	−0.89	0.024
	*GSTA4*	−0.88	0.026
	*RAB4A*	−0.29	0.026
	*CYTH3*	0.52	0.026
	*HSD17B6*	−0.36	0.028
	*RAC1*	0.35	0.038
HF.ZG	*INPP1*	0.73	0.005
	*ALAS1*	−0.94	0.074
HF.H2	*UOX*	0.85	0.028
	*C1R*	7.45	0.055
	*LOC100295234*	2.92	0.055
	*SNCA*	−3.61	0.055
	*FBP2*	−3.06	0.055

*RFI* residual feed intake, *HF* Holstein-Friesian, *H1* high concentrate diet 1, *H2* high concentrate diet 2, *ZG* zero-grazed grass diet, *LogFC* log_2_fold-change in low-RFI steers compared to high-RFI steers; *P*-value = Benjamini-Hochberg corrected *P*-value to account for multiple testing

In CH cattle, three DEGs were common to the H1 and ZG diets, while no gene was common to all three diets. These genes shared between H1 and ZG in CH were *growth arrest and DNA damage inducible gamma* (*GADD45G)*, *haptoglobin precursor* (*HP*) and *MID1 interacting protein 1* (*MID1IP1*). *HP* was upregulated in low RFI steers across both diets, while *MID1IP1* was downregulated in the same diets. However, relative to high RFI, *GADD45G* was upregulated in low RFI CH steers offered the H1 diet, while it was downregulated in low RFI steers offered the ZG diet. There were no common DEGs across dietary phases for HF cattle. Similarly, no DEG was shared across breeds, for any of the three dietary phases.

### Pathway analysis and functional enrichment

For the CH cohort 141 and 143 genes mapped to the Ingenuity Pathway analysis (IPA) knowledge database for the H1 and ZG, respectively. For the HF steers, 26 genes mapped to the H1 diet.

Following IPA analysis, 103 and 77 significantly enriched (Fisher’s exact *P-*value < 0.05) canonical pathways were identified for H1 and ZG, respectively in the CH cohort. Table 5 illustrates the top ten canonical pathways affected by RFI divergence between CH steers for the both diets examined via IPA. For the HF steers offered the H1 diet, 27 significantly enriched canonical pathways were identified. Table 6 lists the top ten canonical pathways for the HF-H1 breed-diet combination, while all enriched canonical pathways are listed in Additional file 3. A total of two pathways were enriched across both examined diets for CH (Table 7). The two pathways for the CH cohort were interlukin-6 (IL-6) signalling and acute phase response signalling.

**Table 5:** The top ten canonical pathways for Charolais steers within each dietary phase for which IPA was performed

Diet-Breed Combination	Canoncial pathway	Differentially Expressed Genes	*P*-value
CH.H1	Toll-like Receptor Signalling	*IL1A, JUN,* ***MAP2K6*** *, NFKBIA, TNFAIP3,* ***UBA52***	8.71E-09
	CD40 Signalling	*IRS2, JUN,* ***MAP2K6*** *, NFKBIA, TNFAIP3*	0.00015
	IL-6 Signalling	**CSNK2B**, IL1A, IRS, JUN, **MAP2K6**, NFKBIA	0.00017
	Aryl Hydrocarbon Signalling	**ALDH9A1**, IL1A, JUN, MYC, TFDP1, **TGM2**	0.00028
	Cholecystokinin/Gastrin mediated Signalling	IL1A, JUN, **MAP2K6**, *MAPK7,* ***RND3***	0.00046
	p53 Signalling	*GADD45G, IRS2, JUN, TNFRSF10A, TP53INP1*	0.00071
	TNFR2 Signalling	*JUN, NFKBIA, TNFAIP3*	0.00093
	Acute Phase Response Signalling	*HP, IL1A, JUN,* ***MAP2K6*** *, NFKBIA,* ***SAA1***	0.00097
	IL-10 Signalling	*IL1A, JUN,* ***MAP2K6*** *, NFKBIA*	0.001
	NFKB Signalling	***CSNK2B,*** *IL1A, IRS2,* ***MAP2K6*** *, NFKBIA, TNFAIP3*	0.0013
CH.ZG	Glycine Betaine Degradation	*BHMT2, DMGDH,* ***SARDH***	0.000029
	Acute Phase Response Signalling	*C5, FGG, HP,* ***HRAS*** *, LBP, SERPINA3*	0.0009
	Hereditary Breast Cancer Signalling	***CCND1*** *,* ***FGFR3*** *,* ***GADD45G*** *,* ***HDAC5*** *,* ***HRAS***	0.0027
	EIF2 Signalling	***ATF5*** *,* ***CCND1*** *,* ***EIF1*** *,* ***FGFR3*** *,* ***FGFR3*** *,* ***HRAS*** *,* ***RPL13***	0.0033
	Role of Macrophages, Fibroblasts and Endothelial Cells in Rheumatoid Arthritis	*C5,* ***CCND1*** *,* ***FGFR3*** *,* ***HRAS*** *,* ***IL17RC*** *,* ***MIF*** *,* ***TRAF4***	0.0043
	Extrinsic Prothombin Activation Pathway	*F5, FGG*	0.0045
	Chronic Myeloid Leukemia Signalling	***CCND1*** *,* ***FGFR3*** *,* ***HDAC5*** *,* ***HRAS***	0.0056
	Germ Cell-Sertoli Junction Signalling	***BCAR1*** *,* ***FGFR3*** *,* ***HRAS*** *,* ***TUBA4A*** *,* ***TUBB4B***	0.0057
	Methylglyoxal Degradation VI	***LDHD***	0.0063
	GADD45 Signalling	***CCND1, GADD45G***	0.0063

*H1* high concentrate, phase 1, *ZG* Zero-grazed grass, *CH* Charolais; *P*-value = Fisher’s exact test *P*-value, bold text indicates gene downregulation in low-RFI steers

**Table 6:** The top ten canonical pathways for Holstein-Friesian steers offered the high-concentrate one diet

HF.H1	Glutathione-mediated Detoxification	*ANPEP,* ***GSTA4*** *,* ***GSTM1***	4.92E-08
	2-amino-3-carboxymuconate Semialdehyde Degradation to Glutaryl-CoA	***ACMSD***	1.693E-05
	Branched-chain α-keto acid Dehydrogenase Complex	***DLD***	0.00016
	2-ketoglutarate Dehydrogenase Complex	***DLD***	0.00024
	2-oxobutanoate Degradation I	***DLD***	0.00024
	Glycine Cleavage Complex	***DLD***	0.00034
	Acetyl-CoA Biosynthesis I (Pyruvate Dehydrogenase Complex)	***DLD***	0.00045
	Fc Epsilon RI Signalling	*FCER1A, RAC1*	0.00052
	Phagosome Formation	*FCER1A, RAC1*	0.00069
	Aryl Hydrocarbon Receptor Signaling	***GSTM1, GSTA4***	0.00076

*H1* high concentrate, phase 1, *HF* Holstein-Friesian; *P*-value = Fisher’s exact test *P*-value, bold text indicates gene downregulation in low-RFI steers

**Table 7:** The canonical pathways shared across dietary phases for which IPA was performed for Charolais steers

Canonical pathway	H1 *P*-value	ZG *P*-value
IL-6 Signalling	0.00017	0.0085
Acute phase response signalling	0.00076	0.00071

*H1* high concentrate, phase 1, *ZG* zero-grazed grass, *CH* Charolais; *P*-value = Fisher’s exact test *P-*value

Following IPA analysis, 14 enriched (Fisher’s exact *P-*value < 0.05) biological functions were significant across all examined dietary phases for both low-RFI CH and HF (Additional file 4).

### Discussion

In order to identify genes associated with RFI status which are not subject to environmental influences, we carried out RNA-Seq on the liver transcriptome of CH and HF steers divergent for RFI across three dietary phases, on a breed-by-breed basis. This analysis identified two biological pathways significantly enriched across all dietary phases for CH steers. Both of these pathways are immune function related. At the individual gene level, we found three DEGs common to two diets within the CH breed. We also identified genes implicated in processes previously associated with variation in RFI such as oxidative phosphorylation and extracellular matrix organisation [25, 27]. The absence of consistently differentially expressed genes within RFI groups across dietary phase and breed supports the previously observed re-ranking of cattle when offered different diets [17, 28].

### Immune function

Two immune related pathways were enriched for genes differentially expressed in CH steers offered the H1 and ZG diets. For the CH steers, the IL-6 signalling pathway was significantly enriched across all investigated diets. This pathway is activated when IL-6 is released from cells of the immune system in response to inflammatory conditions [29]. The second enriched pathway in CH, the acute phase response pathway, is an early step in fighting infection and serves to initiate inflammation upon the detection of pathogens or injury [30]. In support of the immune-related findings in CH, nine of the ten canonical pathways enriched across all diet-breed comparisons for HF steers were also related to immune function or autoimmunity. Previous work by Salleh et al.*,* (2017) reported similar findings whereby they observed that pathways related to immune function were enriched in RFI divergent dairy cattle [31]. In beef cattle, several studies have reported enrichment of immune-related pathways in RFI-divergent cattle [32, 33]. These results coupled with the findings of the current study highlight the role of the immune system in efficient feed usage.

Of the individual genes identified as differentially expressed in more than one diet, *GADD45G* and *HP* have been identified as associated with immune-related functions. In the present study, *GADD45G* expression was upregulated in low RFI steers offered the H1 diet, while its expression was downregulated in low RFI CH steers fed the ZG diet. The increased expression of *GADD45G* in low-RFI steers offered a high-concentrate diet, which is different from previous observations where *GADD45G* was downregulated when Nellore cattle were offered a forage-based diet [23]. However, it has been suggested that liver inflammation may occur when an animal is fed a high-concentrate diet [23, 34]. This may account for the increased expression of *GADD45G* during the H1 phase. The downregulation of *GADD45G* in low-RFI steers offered the ZG diet may indicate that low-RFI steers experience less inflammation than their high-RFI counterparts when offered a grass diet. Previous work in Canadian cattle identified *HP* as downregulated in the liver of low-RFI Angus steers offered a high-concentrate diet [26], however in the present study *HP* expression was increased in low-RFI CH steers offered both the H1 and ZG diets.

Several groups have suggested that increased inflammation leads to poor feed efficiency due to increased energy expended fighting infection, or other pro-inflammatory challenges [35, 36]. However, others have suggested that increased expression of pro-inflammatory genes enables cattle to respond more efficiently to immune challenges and therefore use less energy combating chronic infection [33, 37]. Our results indicate that diet may also play a key role in the effect of the immune system on RFI status by causing a genotype-by-environment interaction, whereby feed type causes inflammation or immune challenge.

### Lipid metabolism

The final DEG identified in both H1 and ZG diets for CH was *MID1IP1,* a gene required for fatty acid and lipid synthesis [38]. *MID1IP1* was observed to be downregulated in low-RFI CH steers offered H1 and ZG diets*.* Downregulation of *MID1IP1* in low-RFI CH cattle is in agreement with previous work carried out in Canadian beef cattle, where it was observed that low-RFI steers displayed lower levels of hepatic lipid synthesis than high-RFI steers [26]. The same authors suggested that decreased lipid synthesis may be due to efficient cattle partitioning greater energy to muscle deposition than fat. Lipid metabolism was also observed to be an enriched biological function in all breed-diet comparisons in this study, illustrating the integral role that this pathway plays in RFI divergence as has been previously reported in other hepatic transcriptome studies [22, 23, 26, 27].

*Diacylglycerol acyltransferase (DGAT)*, a gene within the lipid metabolism biological function, was identified as downregulated in low-RFI CH steers offered the H1 diet. Similarly, Salleh et al. (2017) found this gene to be down-regulated in the hepatic transcriptome of low-RFI Holstein cattle [31]. Contrastingly, *insulin receptor substrate 2 (IRS2)* was observed to be upregulated in low-RFI CH steers offered the H1 diet. Previous work in pigs also observed the upregulation of *IRS2* in feed efficient animals [39]. *IRS2* knockout mice display increased adiposity and total body fat mass [40]. This potentially indicates that downregulation of *IRS2* observed in the high-RFI CH steers offered the H1 diet may lead to increased energy partitioned to fat deposition. *Agouti signalling protein (ASIP)* and *synuclein alpha (SNCA)* were both downregulated in the hepatic transcriptome of CH steers offered the H2 diet. Both of these genes have previously been associated with increased lipid synthesis [41, 42]. These results further support the hypothesis that feed efficient cattle expend less energy for hepatic lipid synthesis than their inefficient counterparts [26]. Efficient cattle may partition more energy to muscle gain than lipid synthesis, and are therefore more feed efficient [26].

### Extracellular matrix proteins

*Tenascin C* (*TNC*) was observed to be upregulated in low-RFI CH steers offered the ZG diet. This gene has previously been identified as upregulated in the liver transcriptome of low-RFI Angus bulls [27]. Those authors hypothesized that the upregulation of *TNC* may indicate that the liver of low-RFI cattle exhibit greater cellular organisation than inefficient cattle. Our results support this hypothesis as we also observed the upregulation of *TNC* in efficient animals and that the biological function cellular assembly and organisation was also enriched in all diet-breed comparisons investigated. Previous work investigating differential gene expression in the rumen epithelium found that *tubulin alpha 4a* (*TUBA4A*) was upregulated in low-RFI crossbred steers [25], further supporting the hypothesis that efficient animals exhibit greater extracellular matrix organisation than their inefficient counterparts. However, we have observed that hepatic *TUBA4A,* was downregulated in the low-RFI steers offered the ZG diet. Consequently, further work is required to elucidate the role of extracellular matrix genes in the liver of RFI-divergent cattle, and the role these genes play in feed efficiency.

### Oxidative phosphorylation

*Glutathione S-transferase Mu 1* (*GSTM1)* encodes for a member of the glutathione S-transferase family. Chen et al.*,* (2011) observed that *GSTM1* was downregulated in low-RFI Angus bulls. These same authors hypothesized that feed efficient cattle experience less oxidative stress and consequently the mRNA abundance of genes involved in the metabolism of oxidative stress products is reduced. Similarly, in the present study, *GSTM1* was identified as a downregulated gene in low-RFI HF steers offered the H1 diet. Our finding of decreased *GSTM1* abundance is in agreement with the hypothesis suggested by Chen et al., (2011) and others who observed that efficient cattle experience less oxidative stress than their inefficient counterparts [27]. This has also been observed in poultry [43, 44]. However, Paradis et al.*,* (2015) and Tizioto et al.*,* (2015) observed that *GSTM1* transcript levels were increased in feed efficient crossbred heifers and Nellore steers, respectively [22, 33]. From their findings, Paradis et al.*,* (2015) suggested that low-RFI cattle respond in a more efficient manner to oxidative stress than their high-RFI counterparts. It is possible that observed variation in *GSTM1* expression across studies may represent a genotype-by-environment interaction whereby certain feed efficient animals experience less oxidative stress, while others may be adapted to deal with this stressor in a more effective manner.

### Effect of differential dietary phases on RFI

The absence of commonly DEGs across all diets for either breed investigated in this study may support previous findings highlighting re-ranking of animals in terms of RFI when they are offered differing diets over their lifetime [17, 28]. These results, as well as the variation in direction of activation of immune genes, such as *GADD45G*, and oxidative stress response genes, e.g. *GSTM1,* across dietary phases highlights the previous suggestions that diet effects RFI status [18]. However, further work is required to validate this hypothesis in larger sample sizes.

Furthermore, an additional method of analysis which may identify genes consistently differentially expressed across breeds within dietary phase would be to conduct analysis in both breeds simultaneously, rather than independently as was the case in this study. This would facilitate identification of genes associated with RFI regardless of breed.

## Conclusion

We investigated differential gene expression using RNA-Seq analysis in the liver of CH and HF steers divergent in RFI across three dietary stages, with the goal of identifying genes and pathways associated with RFI across breed and diet. We identified three DEGs shared across two diets for CH steers. Fourteen biological pathways were shared across all diets which were subjected to IPA for both breeds. The identification of physiological processes such as the immune response as enriched for genes implicated in RFI highlights the importance of this biological process in feed efficiency. Further work investigating genes within identified pathways may enable discovery of biomarkers for RFI that may be incorporated into genomic-assisted breeding programmes, as well as enhancing our understanding of the underlying biology of variation in the RFI trait. However, further work is required in this area in order to replicate and validate these results in independent and larger cattle populations.

## Methods

### Animal model

All procedures involving animals in this study were reviewed and approved by the Teagasc animal ethics committee and were conducted under an experimental licence issued by the Health Products Regulatory Authority (AE19132/P029), in accordance with the Cruelty to Animals Act 1876 and the European Communities (Amendment of Cruelty to Animals Act 1876) Regulations 2002 and 2005.

**Fig. 1:**
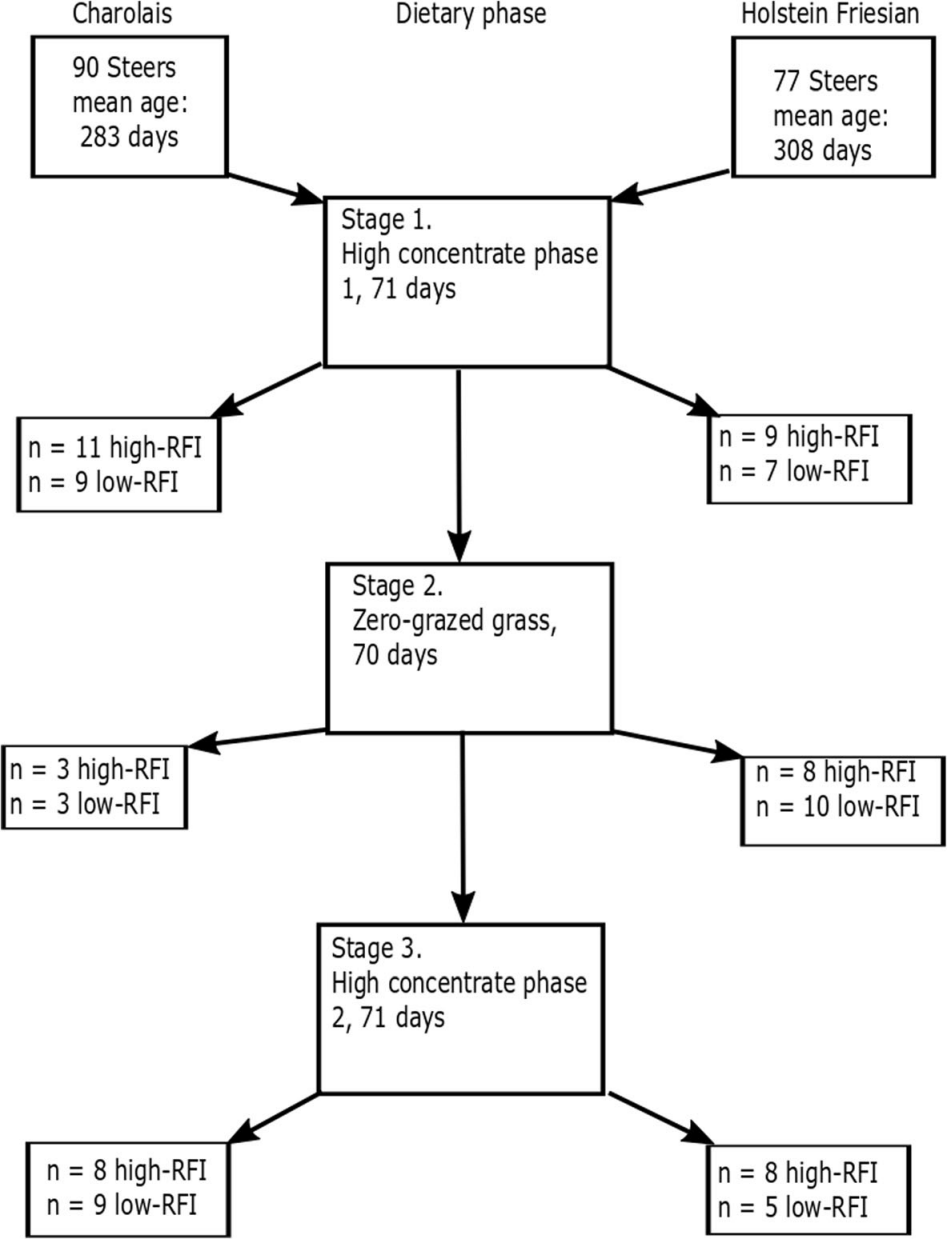
Outline of the feeding trial design during which RFI was measured. During each dietary stage, steers were offered the respective diet for 70 days following a period of dietary adaptation. At the end of each dietary stage, liver biopsies were taken and RFI was calculated. Within breed, all steers were ranked for RFI. RNA-Seq libraries were generated from biopsies taken from the most RFI-divergent steers (*n* = 12 high and *n* = 12 low)

This experiment was conducted as part of a larger study examining genotype-by-environment interactions for and repeatability of feed efficiency across growing and finishing stages of beef production, during which diets offered differed in energy density and chemical composition. The animal model used was described in detail previously [17, 28]. The animals used in this study were purchased from commercial herds and maintained solely for the purposes described in the studies of Coyle et al. [17, 28] and the current study. The experimental design is outlined in Fig. 1. Following the study they were slaughtered in an EU licenced abattoir.

Briefly, 90 CH and 77 HF steers were offered different diets throughout their lifespan. All cattle were initially offered H1 in the growing phase, ZG diet during the growing phase and then H2 during the finishing phase (Fig. 1). Between the H1 and ZG phases cattle were offered a grass silage diet, and between phases ZG and H2 cattle were allowed a grazed grass diet. During these grazed grass and grass silage diets, biopsies were not taken, and data obtained were not included in any analysis pertaining to this work. Individual dry matter intake (DMI) and growth were measured over the three individual feeding phases this study is focussing on, each at least 70 days in duration, which were preceded by dietary adaption periods. During these phases individual feed intake values were measured for each steer daily using a Calan gate system (American Calan Inc., Northwood, NH). At the start of the first dietary phase (H1) the mean age (standard deviation) of the steers was 283 days (18.3) and 306 days (7.7), for CH and HF, respectively. During each individual feeding phase the health of all cattle was monitored. Any animal which required treatment was noted and excluded from downstream analysis.

During H1 and H2, steers were individually offered the same high-concentrate diet ad libitum and a restricted allowance of grass silage daily in order to maintain healthy rumen function. The high-concentrate diet consisted of 860 g/kg rolled barley, 60 g/kg soya bean meal, 60 g/kg molasses and 20 g/kg minerals and vitamins. During the ZG phase, steers were individually offered ad libitum zero-grazed grass (DM 183 g/kg). Grass was harvested twice daily from *Lolium perenne* dominant swards using a zero-grazer. Chemical composition of these diets is as outlined in Additional file 1 [16, 34]. Cattle were given unrestricted access to fresh, clean drinking water throughout all phases of this study.

Steer body weight (BW) was measured, prior to feeding, on at 14-day intervals throughout the dietary phases as well as on two consecutive days at the beginning and the end of each phase. The two measurements taking at the start and end of each phase were averaged in order to get the most accurate starting and finishing weight of each animal, respectively.

### Computation of traits

At the end of each dietary phase, ADG of individual steers was calculated as the coefficient of the linear regression of BW (kg) on time (days) using the GLM procedure of SAS 9.3 (SAS Inst. INC., Cary, NC, USA). Mid-test metabolic weight was computed as BW^0.75^ halfway through each test period, which was estimated from the intercept and the slope of the regression line through all BW^0.75^ observations.

Predicted DMI was computed for each steer, within breed, by regressing DMI on MBW and ADG using a multiple regression model. The model used to compute predicted DMI was:

$$ {Y}_j={\beta}_0+{\beta}_1{MBW}_j+{\beta}_2{ADG}_j+{e}_j, $$

where Y_j_ was the average DMI of the *j*th steer, β_0_ is the regression intercept, β_1_ is the partial regression coefficient on MBW, β_2_ is the partial regression coefficient on ADG and e_j_ is the random error associated with the *j*th animal. RFI was calculated as the difference between actual and predicted DMI. Steers were ranked by RFI within breed for each dietary phase, and the twelve most efficient (low RFI) and the twelve least efficient (high RFI) animals were identified for each breed and phase, and biopsies from these animals were used for RNA-Seq library generation.

### Sample collection, RNA extraction and cDNA library synthesis

Liver tissue was collected from all animals at the end of each dietary phase by percutaneous punch as described by McCarthy et al. (2009) [45]. Animals received local anaesthetic (5 ml Adrenacaine, Norbrook Laboratories, Ireland Ltd.) and care was taken to ensure samples were consistently harvested from the same location for each animal. All instruments used for biopsy collection were sterilized, washed with 70% ethanol and treated with RNaseZap (Ambion, Applera Ireland, Dublin, Ireland). All samples were washed in sterile DPBS, snap frozen in liquid nitrogen and stored at − 80 °C prior to further analysis.

Fifty mg of the biopsied tissue was used for the isolation of total RNA. Samples were homogenised using a rotor-strator tissue lyser (Qiagen, UK) in 3 ml of QIAzol (Qiagen, UK). RNA was extracted and purified using the RNeasy plus Universal kit (Qiagen, UK) as per the manufacturer’s instructions. RNA quantity was determined using a Nanodrop spectrophotometer (Nanodrop Technologies, Wilmington, DE, USA). Quality control checks were carried out on isolated RNA using the RNA 6000 RNA Nano Lab Chip Kit and the Agilent Bioanalyser 2100 (Agilent Technologies Ireland Ltd., Dublin, Ireland). Samples displaying a RNA integrity number of greater than 8 were deemed of sufficient quality for analysis, and were subjected to cDNA synthesis.

cDNA libraries were prepared for sequencing using the Illumina TruSeq stranded mRNA sample prep kit (Illumina, San Diego, CA, USA) as per manufacturer’s instructions. Library validation was conducted using the DNA 1000 Nano Lab Chip which was read using the Agilent Bioanalyser 2100 (Agilent Technologies Ltd. Dublin, Ireland). Library concentration was assessed using a Nanodrop Spectrophotometer (Nanodrop Technologies, Wilmington, DE, USA). Samples with a DNA concentration of greater than 25 ng/μl were subjected to further analysis. Libraries were pooled and 50 base-pair, single-end sequencing was conducted using an Illumina HiSeq 2500. Prior to library generation, some samples were excluded due to poor RNA quality. A total of 45 CH and 58 HF libraries were sequenced successfully (Fig. 1). All sequence data generated as part of this study has been submitted to the Gene Expression Omnibus repository and can be accessed using the accession number GSE111464.

### RNA-Seq data analysis

Sequencing data was supplied in FASTQ format. Adapter and low quality sequence data was removed using cutadapt (v. 1.13) [46]. Reads were retained if they had a base quality of at least 30 and a minimum length of 20 bp. FastQC (v. 0.11.5) [47] was used for quality assessment of the filtered data. Both cutadapt and FastQC were called using TrimGalore! (v.0.4.3) [48]. After trimming, libraries with less than 10^7^ reads were discarded. Before filtering an average of 29.05 million reads per sample was generated, and these reads had an average GC-content of 47.99% with 96.92% of bases having a quality score greater than 30. Following filtering, average reads per sample remained at 29.05 million, and average GC-content increased to 48.06%. Post-filtering, 99.05% of bases had a Q score greater than 30.

Reads were mapped to the bovine reference genome (UMD3.1) [49] using STAR (v.2.5.1) [50]. Protein coding genes were supplied from the Ensembl [51] version 87 annotation of the *Bos Taurus* genome [49]. The STAR parameter “*quantMode GeneCounts”* was used to quantify the mapped reads at the gene level.

Analysis of the gene count data was carried out using the Bioconductor [52] package DESeq2 [53] (v. 1.16.1). Raw gene counts were provided to DESeq2 and an analysis pipeline, DESeq, was applied to the data to accurately calculate dataset-specific analysis parameters and apply negative binomial GLM fitting for use in the subsequent differential expression analysis. Any samples identified as outliers were removed. Low count reads were removed within the DESeq pipeline using the command “*results()*” which removed lowly expressed genes from analysis [53]. The differential expression analyses were performed separately for each breed and each dietary phase where RFI status was fitted as a variable. For each pair of experimental groups under investigation, a list of differentially expressed genes (DEGs) was extracted directly from the DESeq2 data. A Benjamini-Hochberg correction was applied to account for multiple test burden [54]. Following correction, an adjusted *P*-value of < 0.1, the recommended threshold for DESeq2, was used to denote significance.

### Pathway and functional enrichment analysis

Each list of DEGs was further investigated using Ingenuity Pathway Analysis (IPA; Ingenuity Systems, Redwood City, CA, USA). DEGs, along with their respective fold-changes and adjusted *P-*values were submitted to IPA for analysis. Ingenuity pathway analysis allows examination of over-represented biological pathways and biological functions [39]. Ingenuity pathway core analysis was performed on genes identified as statistically significant (adjusted *P* < 0.1) following DESeq2 analysis. However, if too few genes reached an adjusted *P*-value < 0.1 within a diet-breed combination for IPA to be performed, that combination would be excluded from IPA. Consequently, 160 and 158 genes were uploaded to IPA for the CH H1, ZG and H2 diets, respectively, while 27 genes were uploaded to IPA for the HF H1, diet.

Genes were then mapped to IPA biological functions and canonical pathways. Biological functions and canonical pathways were significantly enriched if the *P-*value of the overlap between the input gene list and the genes within the database for a given function or pathway was less than 0.05. Upregulation or downregulation of functions or pathways was determined by a z-score, as calculated by IPA from the expression levels of input genes in a function or pathway. A negative z-score represented downregulation of a function or pathway, while a positive z-score represented upregulation.

## Additional files

Additional file 1: Chemical composition of the feed offered to steers offered three different diets. (XLSX 11 kb)

Additional file 2: All genes deemed to be differentially expressed for each individual dietary phase for Charolias and Holstein-Friesian steers. (XLSX 30 kb)

Additional file 3: All canonical pathways identified as significantly enriched by IPA across each dietary phase for both Holstein-Friesian and Charolais steers. (XLSX 15 kb)

Additional file 4: The range of P-values for the biological functions that were significantly enriched across all dietary phases for low-RFI Charolais and Holstein-Friesian steers. (XLSX 10 kb)

## Data Availability

The datasets generated and analysed in the current study are available in the Gene Expression Omnibus (GEO) repository, and are accessible through the GEO accession number GSE111464.

## Abbreviations

ADG: Average daily gain
BW: Body weight
cDNA: Complementary DNA
CH: Charolais
DEG: Differentially expressed gene
DMI: Dry matter intake
DNA: Deoxyribonucleic acid
GEBVs: Genomic estimated breeding values
H1: High-concentrate phase 1
H2: qHigh concentrate phase 2
HF: Holstein-Frieisan
IPA: Ingenuity pathway analysis
MBW: Metabolic body weight
PCR: Polymerase chain reaction
RFI: Residual feed intake
RNA-Seq: RNA Sequencing
ssGWAS: Single-step genome-wide association study
ZG: Zero-grased grass
